# *MaCDSP32* From Mulberry Enhances Resilience Post-drought by Regulating Antioxidant Activity and the Osmotic Content in Transgenic Tobacco

**DOI:** 10.3389/fpls.2020.00419

**Published:** 2020-04-16

**Authors:** Hongmei Sun, Wenrui Zhao, Hui Liu, Chao Su, Yonghua Qian, Feng Jiao

**Affiliations:** Institute of Sericulture and Silk, College of Animal Science and Technology, Northwest A&F University, Yangling, China

**Keywords:** mulberry *MaCDSP32*, transgenic tobacco, abiotic stress, resilience, antioxidant activity, osmotic accumulation

## Abstract

Desiccation tolerance is a complex phenomenon that depends on the regulated expression of numerous genes during dehydration and subsequent rehydration. Our previous study identified a chloroplast drought-induced stress protein (MaCDSP32) in mulberry, a thioredoxin (Trx) that is upregulated under drought conditions and is likely to confer drought tolerance to transgenic plants. Mulberry (*Morus* spp.) is an ecologically and economically important perennial woody plant that is widely used in forest management to combat desertification. However, its stress tolerance physiology is not well understood. In this study, the functions of *MaCDSP32* gene were investigated. The expression of *MaCDSP32* exhibited a circadian rhythm and was induced by mild and severe water deficits. Under abiotic stress, *MaCDSP32*-overexpressing plants exhibited increased stress sensitivity with lower water retention capacity and more severe lipid peroxidation than the wild-type (WT) plants. Furthermore, the activity of superoxide dismutase (SOD), the contents of proline and soluble sugars and the expression of stress-related transcription factors were lower in the *MaCDSP32*-overexpressing plants than in the WT plants. However, the *MaCDSP32*-overexpressing lines exhibited stronger recovery capability after rewatering post-drought. Moreover, the SOD enzyme activity, proline content, and soluble sugar content were higher in the transgenic plants after rewatering than in the WT plants. The production of the reactive oxygen species (ROS) H_2_O_2_ and O_2_^–^ was significantly lower in the transgenic plants than in the WT plants. In addition, under abiotic stress, the *MaCDSP32*-overexpressing lines exhibited improved seed germination and seedling growth, these effects were regulated by a positive redox reaction involving *MaCDSP32* and one of its targets. In summary, this study indicated that *MaCDSP32* from mulberry regulates plant drought tolerance and ROS homeostasis mainly by controlling SOD enzyme activity and proline and soluble sugar concentrations and that this control might trigger the stress response during seed germination and plant growth. Overall, *MaCDSP32* exerts pleiotropic effects on the stress response and stress recovery in plants.

## Introduction

Plants, as sessile organisms, are constantly facing challenges from their surroundings and have thus evolved a set of precise defense mechanisms and self-repair systems during the long process of survival of the fittest (Zhu, 2016). Reactive oxygen species (ROS), as an inevitable metabolite produced during plant growth or due to environmental hazards, play integral roles as signaling molecules in numerous biological processes but can also cause oxidative damage to cells (Baxter et al., 2014). Excess ROS cause biochemical changes, such as protein unfolding, denaturation or aggregation, which results in the inactivation of various enzymes, and these effects are mostly ascribed to changes in intermolecular disulfide bonds (Rey et al., 1998). However, it is important to maintain the normal physiological functions of some response proteins in plants under stress (Vieira Dos Santos and Rey, 2006). ROS accumulation within chloroplasts is controlled by a complex antioxidant-scavenging system that includes thioredoxins (Trxs) and 2-Cys peroxiredoxins (Prxs), as well as antioxidant enzymes, such as superoxide dismutase (SOD) and ascorbate peroxidase (APX) (Foyer and Shigeoka, 2011). Trxs contain active cysteine residues (Cys-Gly-Pro-Cys), and this disulfide center appears to be highly involved in their function (Vieira Dos Santos et al., 2007; Laugier et al., 2010) by supplying reducing power to the oxidized disulfide of Prxs (Buchanan et al., 2000). Chloroplasts house a large number of different Trx forms. Trx-*x*, Trx-*y* and CDSP32 are Trxs that play important roles in the responses of chloroplast to oxidative stress (Foyer, 2018). Moreover, Trxs reduce the disulfide bridges of target proteins during the Calvin cycle. These Trxs can be reduced by two pathways, which show some overlap with regard to their target proteins: the ferredoxin-dependent Trx system and the NADPH-dependent Trx reductase (NTRC) pathway. These two systems function together with 2-Cys Prxs. The Prx/Trx system is a ubiquitous antioxidant system that removes harmful ROS and is specifically involved in the detoxification of hydrogen peroxide (H_2_O_2_). Trxs modulate the activities of various antioxidant enzymes and the redox status to directly scavenge ROS, and these effects contribute to oxidative stress-linked signaling pathways and ROS homeostasis. Although it is clear that Trxs play a fundamental role in regulating diverse processes in living cells (Montrichard et al., 2009), further studies are needed to determine the specific enzymes targeted by certain Trxs and their interaction regulatory processes in chloroplasts under different abiotic stresses.

Mulberry (*Morus* spp.) is a widely planted economically and ecologically important woody plant species that is used in modern farming, environmental management, and the clothing industry. Its economic value in sericulture, nutritional benefits and medicinal value have received increasing attention (Zeng et al., 2015). In addition, mulberry has worldwide ecological value due to its environmental adaptability, which is reflected in its tolerance to adverse conditions, such as drought, cold, saline-alkali conditions, waterlogging and barren soil (Li et al., 2018). However, the molecular mechanisms involved in tolerance to drought stress are not well understood. Notably, certain species known as resurrection plants have evolved unique mechanisms of desiccation tolerance and can thus tolerate relatively severe water loss. Drought tolerance is a complex phenomenon that depends on the regulated expression of numerous genes during dehydration and subsequent rehydration (Dinakar and Bartels, 2013), but the underlying regulatory mechanisms remain unclear. Thus, an understanding of the mulberry genes involved in responses to adverse conditions should provide useful genetic resources for future molecular plant breeding. In particular, since the announcement of the mulberry genome in 2013, studies of genes related to tolerance in mulberry have been gradually conducted (He et al., 2013). Our preliminary work identified a wide range of differentially expressed proteins in drought-treated mulberry *ShinIchinose* (*Morus alba* L.), and among these proteins, we identified an upregulated protein, denoted mulberry chloroplast drought-induced stress protein (MaCDSP32), which is a plastidial Trx-like protein with two Trx domains (Rey et al., 1998). CDSP32 is involved in both the regeneration of plant Prxs (Dietz, 2007) and catalysis of the thiol-disulfide interchange (Sugiura et al., 2019). A previous study revealed that plants overexpressing *CDSP32* display increased sensitivity to oxidative stress (Broin et al., 2002). However, transgenic potato plants deficient in the CDSP32 protein present higher levels of overoxidized forms of Prx monomers and increased lipid peroxidation (Broin et al., 2003). These results are contradictory, and the reasons remain unclear. The last review of *CDSP32* provided little detailed functional knowledge (Dietz, 2007), but recent studies have shown that *CDSP32* is involved in a variety of physiological and biochemical processes, including the anthocyanin biosynthetic pathway in arctic mustard (Butler et al., 2014) and the cadmium tolerance process in oilseed rape (Zhang et al., 2018). In addition, B-type methionine sulphoxide reductase (MSRB) is a target of CDSP32 (Rey et al., 2005, 2013). Methionine sulphoxide reductase (MSR) regulates the life of seeds, and the metabolites of methionine specifically facilitate seed germination (Catusse et al., 2011; Chatelain et al., 2013). Whether *MaCDSP32* participates in the regulation of seed germination through a mechanism related to MSRB has not been previously reported. Thus, the function of MaCDSP32 in mulberry piqued our interest. In this study, its coding gene, *MaCDSP32*, was transformed into tobacco (*N, benthamiana*) to assess the changes in the various abiotic stress tolerances of the transgenic plants. We found that the ectopic expression of *MaCDSP32* altered the stress signal transduction pathway and the production of ROS under abiotic stress and stress relief conditions. Furthermore, the potential action of *MaCDSP32* in seed germination was evaluated. The aim of this work was to characterize the functions of *MaCDSP32* during exposure to abiotic stress. Overall, our results suggest a novel functional model for Trx MaCDSP32 in plant stress tolerance and post-stress recovery and provide a reference for the exploitation of mulberry germplasm resources.

## Materials and Methods

### Plant Materials and Growth Conditions

Laboratory mulberry plants of the *ShinIchinose* cultivar were produced by tissue culture from winter buds. Laboratory mulberry *Luza* seedlings were grown from seeds. Five-month-old laboratory *ShinIchinose* plants and laboratory *Luza* plants were prepared for use in this study. The plants were grown in a mixed nursery substrate composed of turfy soil and vermiculite. In addition, the mature leaves from the annual shoots of *ShinIchinose* trees grown in the field were used for the expression assay of circadian rhythm gene. The growth of these trees was completely dependent on the natural conditions, without artificial watering. Tobacco (*N. benthamiana*) was used for the generation of overexpression transgenic plants. The laboratory mulberry and tobacco plants were grown in a greenhouse under the controlled conditions (23°C, 16-h light/8-h dark, 50% humidity) and supplied 1/4-strength Hoagland nutrition solution once per week.

### Gene Cloning, Sequencing and Bioinformatics Analysis

Total RNA from the leaves of *ShinIchinose* was extracted using an Ultrapure RNA kit (CWBIO, China) according to the manufacturer’s recommended protocol. First-strand cDNA was synthesized from 1 μg of total RNA using the PrimeScript RT reagent kit with gDNA Eraser (Takara, Japan). The primers used for amplification of the full-length sequence of *MaCDSP32* (the coding gene of mulberry chloroplast drought-induced stress protein, MaCDSP32) (Supplementary Table S1) were designed according to the sequence of *Morus notabilis CDSP32* (XM_010101817.1). PCR was performed using 2 × Taq MasterMix (CWBIO) according to the manufacturer’s recommended protocol. After detection by agarose gel electrophoresis, the PCR product bands were recovered using a gel recovery kit (Omega, United States) and sequenced. Bioinformatics analysis was conducted as described by Wang et al. (2018). The phylogenetic tree was constructed through neighbor-joining analysis using MEGA 6.0.

### Expression Pattern of *MaCDSP32*

To detect the circadian expression patterns of *MaCDSP32*, mature leaf samples from the annual shoots of *ShinIchinose* plants in the field were collected over 3 days (during sunny weather). Leaf samples were collected every 3 h from 06:00 to 21:00 in each day, with three biological replicates per day. To detect the tissue-specific expression patterns of *MaCDSP32*, the leaves (1st, 2nd, 3rd, 4th, 6th, and 9th from the top), petioles and stems from five-month-old laboratory *ShinIchinose* plants were collected at 10:00 under normal water conditions. Three biological replicates were included. Samples were frozen in liquid nitrogen and stored at −80°C.

### Plasmid Construction and Plant Transformation

Full-length *MaCDSP32* was ligated into the pCAM35S-GFP botany vector using a homologous recombination system. The recombinant pCAM35S-*MaCDSP32*-GFP plasmids were transformed into *A. tumefaciens* (GV3101) for plant transformation. Transient transformation was performed in the leaves of *ShinIchinose* according to a vacuum immersion protocol (Matsuo et al., 2016). Three days after transformation, the *MaCDSP32* gene expression in the leaves was confirmed by RT-PCR. The leaves with successful expression were designated *Inst* (*Inst*-1 and *Inst*-2 were selected for subsequent study due to their higher *MaCDSP32* expression), and the wild type was designated WT*_*Ma*_*. Stable transformation was performed in tobacco using a leaf disk co-cultivation protocol (Horsch et al., 1985; Bao et al., 2017). The positive transgenic tobacco lines were selected by 35 mg/ml kanamycin and confirmed by genomic DNA PCR (Rahim et al., 2019). The presence of *MaCDSP32* in each selected transgene was verified by qRT-PCR (La Mantia et al., 2018). The identified positive lines were subcultured until roots formed and then transferred to soil. The tobacco lines with successful overexpression of *MaCDSP32* were named OE lines. The OE-2 and OE-7 lines had higher *MaCDSP32* expression than the other lines and were therefore selected for subsequent experiments. The wild-type tobacco lines were named WT. Seeds from the homozygous transgenic lines were harvested and dried for subsequent use.

### Subcellular Localization

The constructed pCAM35S-*MaCDSP32*-GFP plasmids and the control pCAM35S-GFP vector plasmids were transformed into tobacco (*N. benthamiana*) leaves mediated by *A. tumefaciens* (GV3101) to express fusion proteins with green fluorescence protein (GFP). After 3 d of incubation, the GFP fluorescence in tobacco leaves was imaged using a laser-scanning confocal microscope (TCS SP8 CARS; Leica, Germany). This experiment was performed three times with identical results, and at least six leaves were included in each experimental repeat.

### Abiotic Stress Treatment, Water Loss Rate and Gene Expression

For the drought treatment, 5-month-old *Luza* and *ShinIchinose* plants were subjected to a watering halt for 6 d. Leaves from *Luza* were collected after 0, 1, 2, 3, 4, 5 and 6 d of drought, and leaves from *ShinIchinose* were collected after 0, 3 and 6 d of drought. Three-month-old plants of the OE-2, OE-7 and WT tobacco lines were subjected to drought for 10 d and then rewatered for 4 d. The percentage of wilted leaves in each whole plant at different times points during treatment was recorded. For the NaCl and oxidative stress treatments, three-month-old plants of the OE-2, OE-7 and WT tobacco lines were watered with 200 mM NaCl for 13 d or sprayed with 10 μM methyl viologen (MV) for 25 h according to Li et al. (2009). At least three replicates per treatment were established. All samples were collected at 10:00, frozen in liquid nitrogen and stored at −80°C.

The water loss rate was measured according to Rao et al. (2015). Mulberry leaves (*Inst*-1, *Inst*-2 and WT*_*Ma*_*, at least five leaves per set) were placed on dry filter paper to measure their natural rate dehydration. The water loss rate of tobacco leaves (OE-2, OE-7 and WT, at least five leaves per set) was also measured. All the leaves were weighed every 10 min, for a total of 120 min. The stomatal apertures was measured at 0, 30, and 60 min of dehydration. The expression levels of *MaCDSP32*, the dehydration responsive element binding protein gene (*MaDREB1*) and the mitogen-activated protein kinase gene (*MaMAPK*) in the treated *Inst*-1, *Inst*-2 and WT*_*Ma*_* leaves were examined. Measurements were conducted with at least three replicates.

### Seed Germination and Seedling Growth Under Abiotic Stress

Seeds of the transgenic (OE-2 and OE-7) and WT tobacco lines were germinated on 1/2 MS medium containing mannitol or NaCl at one of two concentrations (100 mM and 200 mM). Medium without additives was used as control medium. The seed germination rate at 5, 6, 7 and 8 d after sowing was scored, and the production of H_2_O_2_ and O_2_^–^ and the gene expression of *MaCDSP32* and *NtMSRB* after 6 d of treatment with 100 mM NaCl or 100 mM mannitol were measured. Nine days after germination under normal conditions, the germinated seedlings of the transgenic (OE-2 and OE-7) and WT tobacco lines were transplanted to 1/2 MS medium containing 200 mM NaCl for an additional 16 d of growth or to medium without additives (control). Seedlings length at initial transplantation and after 16 d of treatment was measured as described by Cui et al. (2019). The gene expression of *MaCDSP32* and *NtMSRB* in treated seedlings was detected after 16 d of growth under stress treatment. At least three replicates were established for each treatment.

### Quantitative Real-Time PCR (qRT-PCR)

qRT-PCR was performed using a TB Green Premix Ex Taq II kit (Tli RNaseH Plus) (Takara) according to the manufacturer’s instructions for gene expression analysis. The template cDNA fragment was synthesized from the extracted total RNA and was diluted to 1/10 for use in the qRT-PCR system. All the primers are detailed in Supplementary Table S1. The procedure was performed with a LightCycler 480II machine (Roche, Switzerland) using a 45-cycle program (95°C for 5 s and 60°C for 30 s). The relative expression of genes was calculated based on the threshold cycle according to the _Δ Δ_ Ct method (Schmittgen and Livak, 2008). The result was normalized to the expression of the reference gene *actin* in mulberry (Chen et al., 2018) or *L25* in tobacco (Liu et al., 2018). Expression was determined for at least three replicates per treatment.

### Quantification of Relative Water Content (RWC)

The RWC of the leaf samples was determined by measuring the fresh leaf weight (FW) and the dry weight (DW) after drying at 65°C for 24 h, as well as the saturated weight (SW) after soaking in water for 1 h, as described by Wang et al. (2015). The following formula was used: RWC (%) = (FW – DW)/(SW – DW) × 100%.

### Measurement of Malondialdehyde (MDA), Proline and Soluble Sugars Contents

The MDA content was detected through the thiobarbituric acid (TBA) reaction as described by Duan et al. (2012). The leaf samples (0.3 g) were homogenized in 5 ml of 50 mM phosphate buffer (pH 7.8) and centrifuged at 10000 × *g* for 10 min. Two milliliters of supernatant was added to 5 ml of 0.5% (w/v) TBA in 10% (w/v) trichloroacetic acid (TCA). The mixture was incubated in boiling water for 10 min, cooled at room temperature and then centrifuged at 3000 × *g* for 10 min. The absorbance of the supernatant was determined at 450 nm, 532 nm, and 600 nm and the following formula was used: MDA (μM⋅g^–^^1^) = 6.45 (A_532_-A_600_) – 0.56A_450_.

The proline content was determined using acid ninhydrin. The leaf samples (0.2 g) were homogenized in 3 ml of 3% aqueous sulfosalicylic acid and centrifuged at 12000 × *g* for 10 min. Subsequently, 500 μl of the supernatant was added to 1 ml of distilled water and 2 ml of 2% ninhydrin in acetone. The mixture was boiled for 15–30 min until it turned pink, cooled, added to 3 ml of toluene, mixed and allowed to stand for 5 min. The absorbance of the upper phase was determined at 520 nm. The proline content in the samples was calculated according to the standard curve and the formula described by Chen et al. (2019).

The soluble sugar content was determined using anthrone. A portion of each sample (0.2 g) was added to 3 ml of 80% ethanol and extracted in a water bath at 80°C for 30 min. Subsequently, 0.1 ml of the extract was added to 3 ml of anthrone reagent (0.2 g of anthrone and 1.0 g of thiourea in 100 ml of concentrated sulfuric acid). The mixture was placed in a boiling water bath for 10 min. The absorbance of the mixture was determined at 620 nm. The soluble sugar content was calculated according to the standard curve and the formula described by Chen et al. (2019). Measurements were obtained for at least three replicates per treatment.

### *In vivo* Localization and Quantification of H_2_O_2_ and O_2_^–^

The *in vivo* detection of H_2_O_2_ and O_2_^–^ was accomplished by histochemical staining with 3,3’-diaminobenzidine (DAB) and nitro blue tetrazolium (NBT) as described by Yadav et al. (2012). The presence of H_2_O_2_ and O_2_^–^ in transgenic and WT leaves exposed to drought and salt stress was detected by immersing the leaf samples in solutions of DAB (1 mg/ml, pH 3.8) and NBT (1 mg/ml) in 10 mM phosphate buffer (pH 7.8). For the detection of O_2_^–^, the leaves were illuminated for 12 h until blue spots, which are indicative of formazan precipitates, appeared. To determine the localization of H_2_O_2_, the immersed leaves were incubated in the presence of light at room temperature for 24 h until brown spots became visible; these spots occurred due to the reaction of DAB with H_2_O_2_. After incubation, the leaf chlorophyll was bleached in absolute ethanol to enable visualization of the blue and brown spots.

The O_2_^–^ content was determined according to the methods described by Yadav et al. (2012). The leaf tissue was extracted in 10 ml of 65 mM potassium phosphate buffer (pH 7.8) and centrifuged at 5000 × *g* for 10 min. A reaction mixture containing 0.9 ml of 65 mM phosphate buffer (pH 7.8), 0.1 ml of 10 mM hydroxylamine hydrochloride, and 1 ml of the extract was incubated at 25°C for 20 min. Subsequently, 17 mM sulphanilamide and 7 mM α-naphthylamine were added, and the mixture was further incubated at 25°C for 20 min. The absorbance was read at 530 nm. A standard curve (10–200 nM) was prepared with NaNO_2_ to calculate the production rate of O_2_^–^.

The H_2_O_2_ content in the leaf samples was measured as described by Yadav et al. (2012). The leaf tissue was extracted with cold acetone. Two milliliters of extract was mixed with 0.5 ml of 0.1% titanium dioxide in 20% (v/v) H_2_SO_4_, and the mixture was centrifuged at 6000 × *g* for 15 min. The absorbance of the supernatant was measured at 415 nm, and the concentration of H_2_O_2_ was calculated based on the established standard curve. Measurements were obtained for at least three replicates.

### Quantification of Antioxidant Activity

The activity of antioxidant enzymes was detected as described by Wang et al. (2008). The leaf samples (0.3 g) were ground in liquid nitrogen and then homogenized in 5 ml of 50 mM phosphate buffer (pH 7.0). The homogenate was centrifuged at 10000 × *g* for 20 min at 4°C, and the supernatant was assayed for the activities of antioxidant enzymes. Protein quantification was determined using a BCA protein assay kit (Jiancheng, China) according to the manufacturer’s recommended protocol, with bovine serum albumin used as the standard. SOD activity was measured with 0.1 ml of supernatant by monitoring the superoxide radical-induced reduction of NBT at 560 nm. One unit of SOD was defined as the amount of enzyme causing 50% inhibition of the reaction compared with the reaction in a blank sample. Catalase (CAT) and APX activities were determined with 0.2 ml of supernatant by following the breakdown of H_2_O_2_ and the ascorbate oxidation at 240 nm and 290 nm over 3 min, respectively. The reaction was initiated by the addition of H_2_O_2_. Peroxidase (POD) activity was measured with 0.2 ml of supernatant at 470 nm, following the oxidation of guaiacol. One unit of enzyme activity was defined as the amount of enzyme causing 50% inhibition per mg of protein and was expressed as U⋅mg^–^^1^ protein. Measurements were obtained for at least three replicates.

### Gas Exchange Parameters and Photosynthetic Pigments Related to Photosynthesis

To evaluate the changes in the photosynthetic system, the gas exchange parameters of the WT and transgenic tobacco lines after 10 d of drought, 13 d of treatment with 200 mM NaCl and 25 h of treatment with 10 μM MV were determined. The gas exchange parameters, including the net photosynthetic rate (*Pn*), stomatal conductance, intercellular CO_2_ concentration, transpiration rate and leaf temperature, were assessed using a portable photosynthetic transpiration system (Yaxin-1102, Yaxin, China) according to the operating instructions. An open-circuit automatic measurement mode was selected. Each measurement was repeated three times, and at least three plants were included in each treatment group.

The chlorophyll and carotenoid contents were measured according to Grzeszczuk et al. (2018) and Rey et al. (2013). The leaf samples were homogenized in 80% acetone and centrifuged at 3000 × *g* for 3 min. The absorbance of the supernatant was recorded at 663 nm, 645 nm and 470 nm. The contents of chlorophyll *a* (*C*_*a*_) and chlorophyll *b* (*C*_*b*_) were determined as follows: *C*_*a*_ = 12.21 × *A*_663_− 2.81 × *A*_645_, *C*_*b*_ = 20.13 × *A*_645_− 5.03 × *A*_663_. The carotenoid content was determined as follows: carotenoid contents = (1000 × *A*_470_− 3.27 × *C*_*a*_ –104 × *C*_*b*_)/229. Measurements were obtained for at least three replicates.

### Statistical Analysis

Statistical analyses were performed using SAS 9.1 and Excel software, and figures were prepared using GraphPad Prism 6.0 software. At least three biological replicates were included in each experiment. The data are presented as the means ± SDs of the biological replicates. The significance of differences was analyzed using Student’s *t*-test. ^∗^*P* < 0.05, ^∗∗^*P* < 0.01 and ^∗∗∗^*P* < 0.001.

## Results

### *MaCDSP32* Cloning, Bioinformatics Analysis and Expression Pattern

**FIGURE 1:**
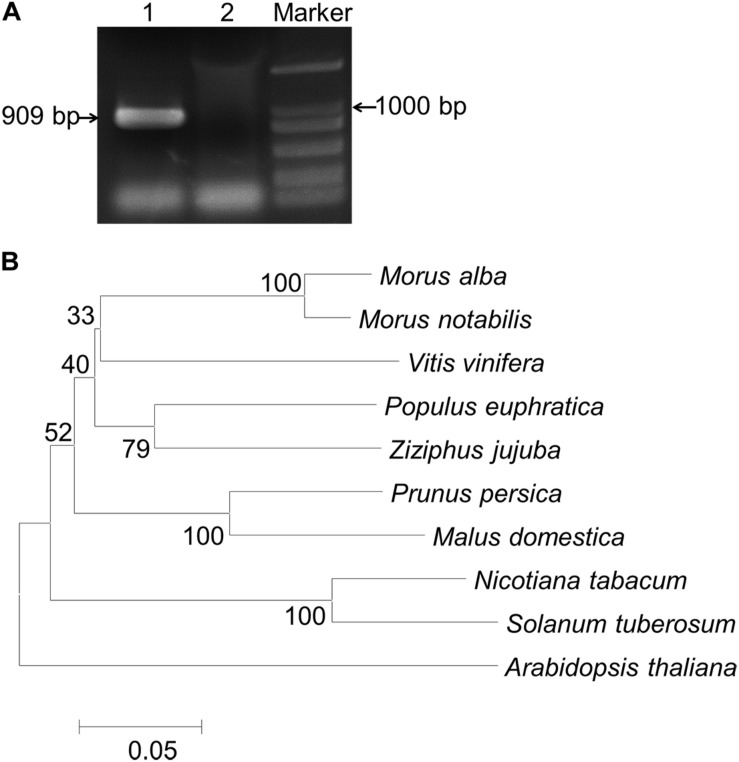
PCR products of *MaCDSP32* and neighbor-joining phylogenetic analysis. **(A)** PCR amplification products of *MaCDSP32*. Lane 1: products of MaCDSP32; Lane 2: negative control (distilled water) used as a template for PCR. **(B)** A phylogenetic tree of the amino acid sequences of CDSP32 proteins from *Rosales* and several typical plant species was constructed using the MEGA 6.0 program.

A full-length gene fragment of *MaCDSP32* was amplified from mulberry *ShinIchinose* by RT-PCR (Figure 1A). The coding region (909 bp) was cloned, sequenced, and submitted to GenBank (accession number: KY799583). The deduced amino acid sequence was 302 amino acids. A phylogenetic tree was constructed using MEGA 6.0 (Figure 1B). The amino acid sequence of MaCDSP32 showed the highest similarity with the protein sequences from *Morus notabilis* (XM 010101817.1), followed by that from *Vitis vinifera*, and the least similarity with the proteins from *Arabidopsis* and *Solanum tuberosum*. Therefore, it is likely that the physiological function of *MaCDSP32* is very different from that known in potato.

**FIGURE 2:**
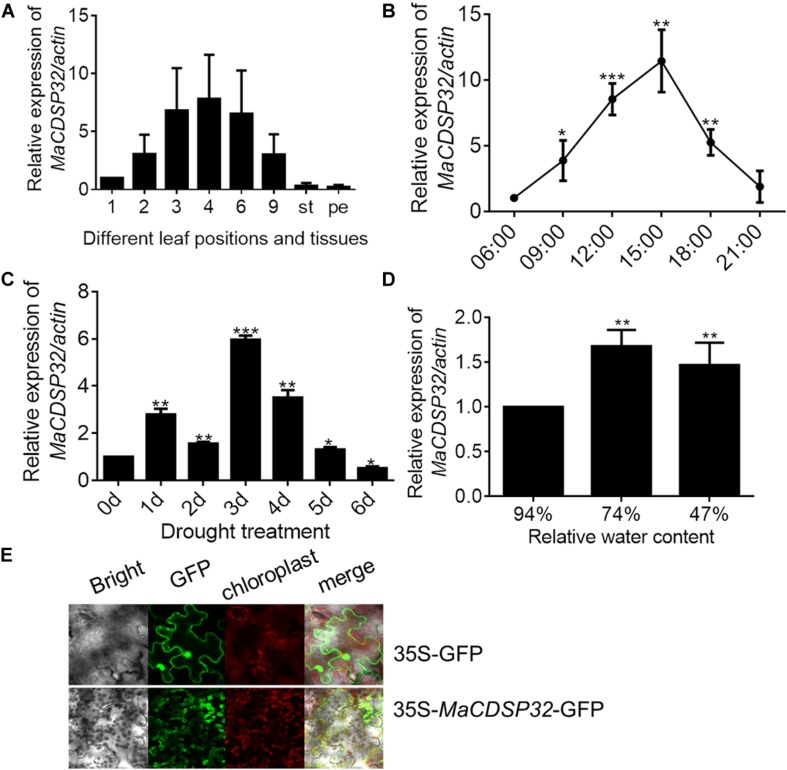
Expression pattern of *MaCDSP32* in mulberry. **(A)** Measurements were obtained from the aboveground parts of the *ShinIchinose* mulberry cultivar; 1, 2, 3, 4, 6, and 9 represent the different leaf positions from top to bottom; st, stem; pe, petiole. **(B)** Measurements obtained within a day. Samples were collected on 3 consecutive days (during sunny weather), with three replicate samples collected per time point. **(C)** Measurements obtained during 6 days of drought in *Luza* cultivar. **(D)** Measurements obtained under different relative water contents (RWCs) of leaves of the *ShinIchinose* cultivar. **(E)** Subcellular localization of MaCDSP32. The control (plasmid with 35S-*GFP* alone) and fusion plasmid (35S-*MaCDSP32-GFP*) are shown. At least three biological replicates were included. Asterisks indicate significant differences between the transgenic and wild-type leaves (*t*-test, *P < 0.05, **P < 0.01, ***P < 0.001).

The gene expression analyses showed that *MaCDSP32* was mainly expressed in mature leaves (of the 3rd, 4th, and 6th leaf positions) rather than in young (1st leaf position) or senescent (9th leaf position) leaves or other tissues, such as stems and petioles in *ShinIchinose* mulberry plants (Figure 2A). Analysis of the expression changes with the circadian rhythm showed that compared to that at 06:00, *MaCDSP32* gene expression was markedly increased at 09:00 and 12:00, peaked at 15:00, and was substantially decreased at 18:00 and 21:00. Moreover, the level at 21:00 was similar to that at 06:00 (Figure 2B). *MaCDSP32* is involved in photosynthesis; accordingly, circadian transcriptional activity was detected. Additionally, the expression products of *MaCDSP32* were localized in chloroplasts (Figure 2E). The results suggest that *MaCDSP32* might function as a clock gene expressed during the day in association with sun exposure to affect chloroplast function. Regarding abiotic stress treatments, *MaCDSP32* expression under drought conditions was detected in the two mulberry cultivars. In the *Luza* cultivar, *MaCDSP32* gene expression was upregulated after 5 d of drought and peaked at 3 d (Figure 2C). In the *ShinIchinose* cultivar, leaf RWC decreased with increasing soil desiccation during the drought treatment (hydrated leaf, 94% RWC); mildly wilted leaf, 74% RWC), severely wilted leaf, 47% RWC). The gene expression of *MaCDSP32* was increased at RWC 74% than that at RWC94%, while was slightly downregulated at RWC 47% than that at 74% (Figure 2D). These results suggest that *MaCDSP32* expression is induced by water deficit but that expression does not increase linearly with drought intensity, declining only slightly under severe water deficit.

### *MaCDSP32* Overexpression Increases Leaf Water Loss Under Dehydration

**FIGURE 3:**
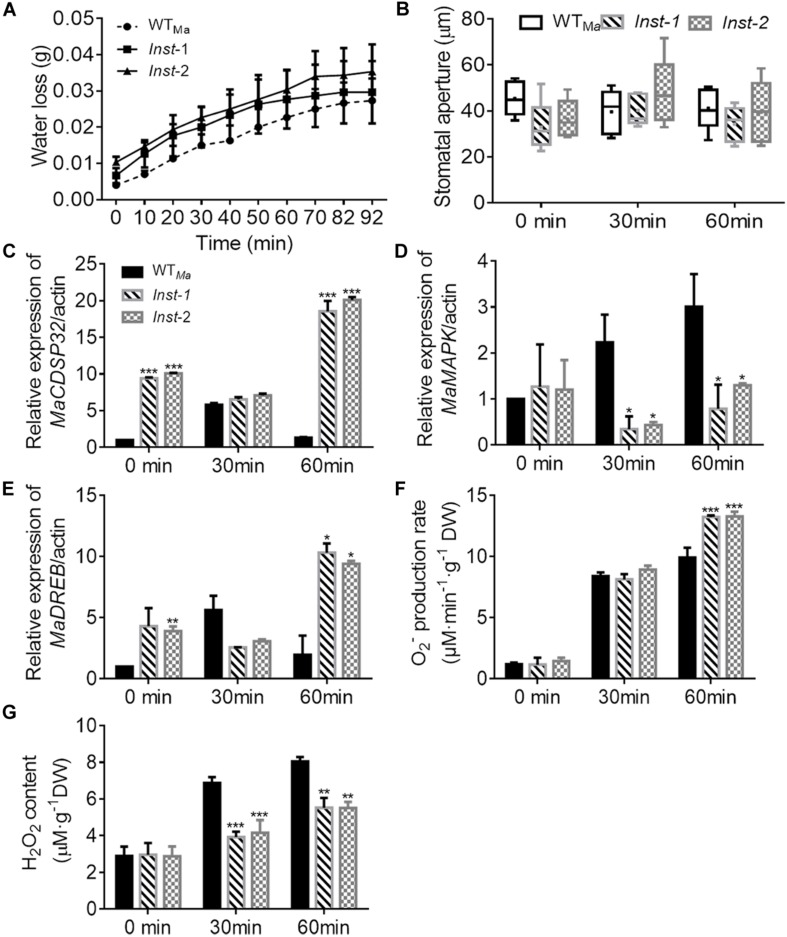
The transient overexpression of *MaCDSP32* increases leaf water loss under desiccation conditions in mulberry. **(A)** Water loss, **(B)** Stomatal aperture after 0, 30, and 60 min of desiccation. **(C–E)** Relative expression of *MaCDSP32* **(C)**, *MaMAPK* **(D)**, and *MaDREB1* **(E)** genes. **(F–G)** Quantification of H_2_O_2_ **(F)** and O_2_^–^ **(G)** contents. WT*_*Ma*_*, leaves of wild-type *ShinIchinose*; *Inst*-1 and *Inst*-2, leaves of *ShinIchinose* transiently overexpressing *MaCDSP32*. At least three biological replicates were included. Asterisks indicate significant differences between the transgenic and wild-type leaves (*t*-test, *P < 0.05, **P < 0.01, ***P < 0.001).

Three days after transient transformation in mulberry leaves, the expression level of *MaCDSP32* in *Inst* (*Inst*-1 and *Inst*-2) and WT*_*Ma*_* leaves was detected. *MaCDSP32* expression was higher in *Inst*-1 and *Inst*-2 than in WT*_*Ma*_* (at 0 min, baseline condition), and upregulated after 60 min under dehydration (Figure 3C). These findings confirmed the effectiveness of the transient transformation, ensuring the reliability of the experiment results. During desiccation treatment, the water loss in *Inst*-1 and *Inst*-2 leaves was higher than that in WT*_*Ma*_* leaves over the 92 min of observation (Figure 3A). Because water evaporation is often related to the stomatal aperture, the changes in the stomatal aperture were measured. The stomatal aperture in *Inst*-1 and *Inst*-2 was not significantly different from that in WT*_*Ma*_* at both baseline (0 min) and during the desiccation treatment (30 and 60 min) (Figure 3B). At 30 min of rehydration, *MaCDSP32* expression was increased in WT*_*Ma*_* but significantly decreased in *Inst*-1 and *Inst*-2; however, after 60 min of rehydration, *MaCDSP32* expression was decreased in WT*_*Ma*_* and markedly increased in *Inst*-1 and *Inst*-2 (Figure 3C). *MaMAPK* expression did not significantly differ between WT*_*Ma*_* and *Inst*-1 or *Inst*-2 at baseline (at 0 min) but was upregulated in WT*_*Ma*_* and downregulated in *Inst*-1 and *Inst*-2 under stress conditions (at 30 and 60 min of dehydration) (Figure 3D). Under stress conditions, *MaDREB1* expression in *Inst*-1 and *Inst*-2 showed the same trends as *MaCDSP32* expression (Figure 3E). Additionally, the production of O_2_^–^ was increased in WT*_*Ma*_*, *Inst*-1 and *Inst*-2 at 30 min of dehydration, with no significant difference among the three leaf types. However, after 60 min of dehydration, O_2_^–^ production was significantly higher in *Inst*-1and *Inst*-2 than in WT*_*Ma*_* (Figure 3F). H_2_O_2_ content did not significantly differ among *Inst*-1, *Inst*-2 and WT*_*Ma*_* under control conditions, but was significantly lower in *Inst*-1 and *Inst*-2 than in WT*_*Ma*_* during dehydration (30 and 60 min) (Figure 3G).

**FIGURE 4:**
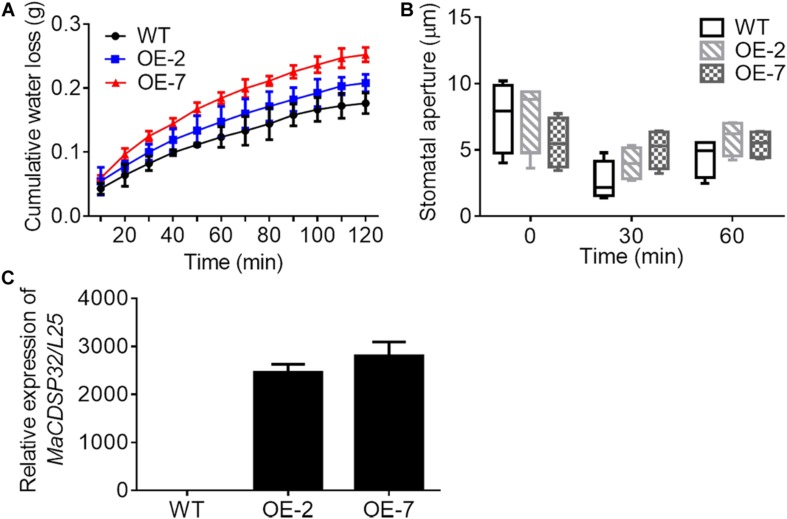
Gene overexpression of *MaCDSP32* increases water loss under dehydration conditions in transgenic tobacco. **(A)** The accumulated water loss of detached leaves under natural desiccation. **(B)** Stomatal aperture of leaves after 0, 30, and 60 min under natural desiccation. **(C)** Detection of *MaCDSP32* gene expression in transgenic tobacco. WT, wild-type tobacco (*N. benthamiana*); OE-2 and OE-7, two transgenic tobacco lines with *MaCDSP32* overexpression. At least three biological replicates were included. No significant difference was observed between the transgenic and WT lines.

Two *MaCDSP32*-overexpression transgenic tobacco lines, OE-2 and OE-7, were selected for exploring the function of the *MaCDSP32* gene (Figure 4C). Detached leaves from the transgenic and WT plants were weighed at regular intervals to record the water loss over the natural drying process. The results showed that the cumulative water loss in the OE-2 and OE-7 lines was higher than that in the WT lines during the dehydration process (Figure 4A). In particular, the OE-7 line, which exhibited the highest *MaCDSP32* expression, showed the greatest water loss. Stomatal aperture in the OE-2 and OE-7 lines was slightly larger than that in the WT lines after 30 and 60 min of dehydration, although the difference was not significant (Figure 4B).

### *MaCDSP32* Attenuates Abiotic Stress Tolerance but Strengthens Resilience After Rewatering

**FIGURE 5:**
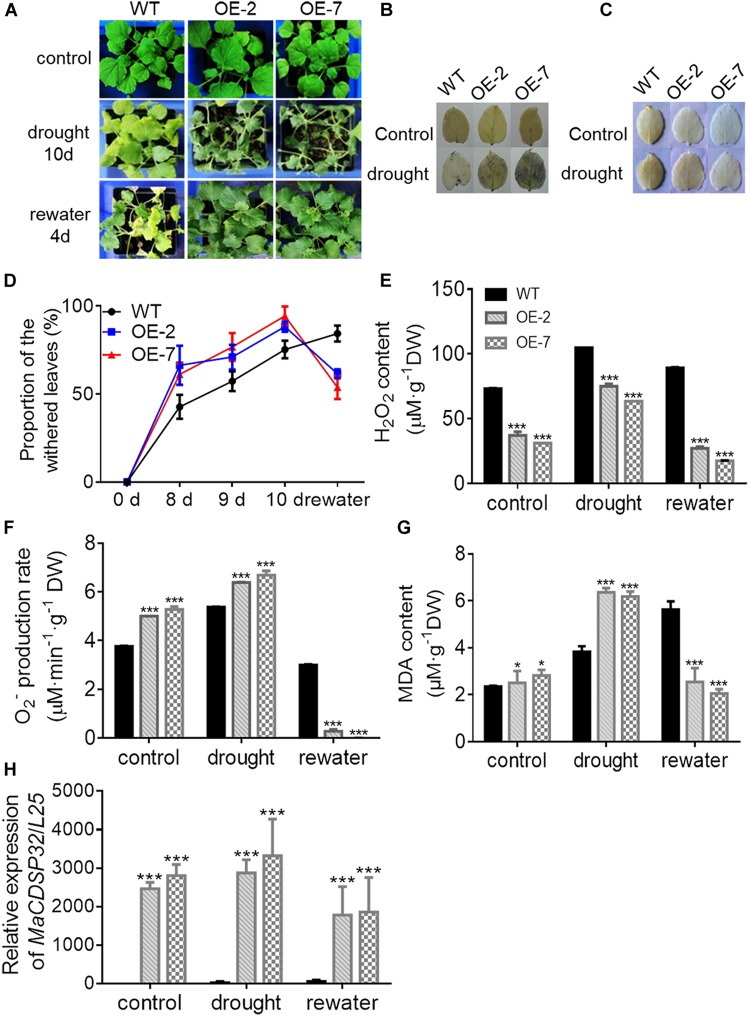
Overexpression of the *MaCDSP32* gene increases drought sensitivity but strengthens recovery during post-drought rewatering in transgenic tobacco. **(A)** Phenotypes of 3-month-old WT plants and two transgenic (OE-2 and OE-7) tobacco lines after 10 days of drought and 4 days of rewatering treatment. **(B,C)** *In vivo* histochemical detection of O_2_^–^ **(B)** and H_2_O_2_ **(C)** after 10 days of drought. **(D)** Proportions of wilted leaves per plant under the treatment. **(E,F)** Quantification of H_2_O_2_ **(E)** and O_2_^–^ **(F)** contents in leaves under the treatment. **(G)** Malondialdehyde (MDA) content in leaves under the treatment. **(H)** Gene expression of *MaCDSP32* under treatment. At least three biological replicates were included. Asterisks indicate significant differences between the transgenic and WT lines (*t*-test, *P < 0.05, ***P < 0.001).

**FIGURE 6:**
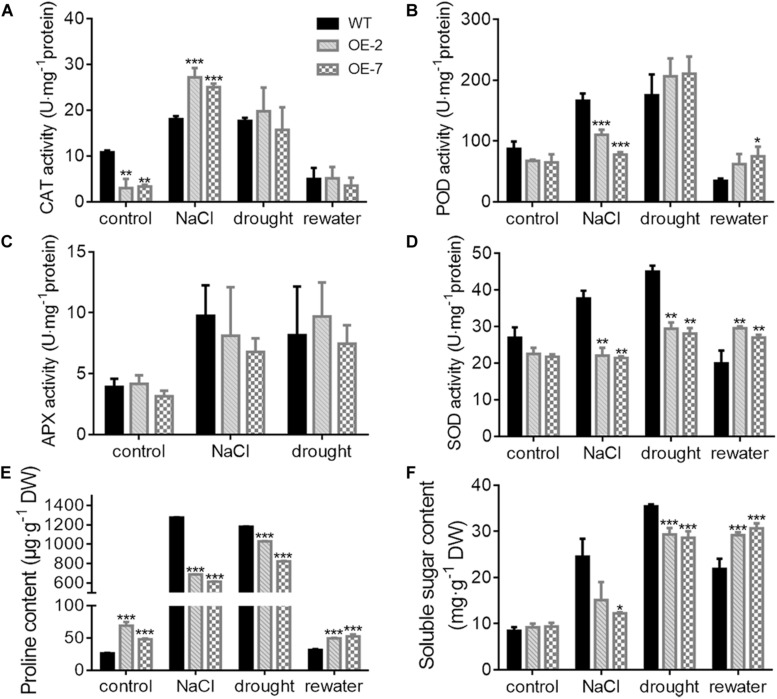
Antioxidant activity and osmotic content in transgenic tobacco under abiotic stress. **(A–D)** Activities of catalase (CAT) **(A)**, peroxidase (POD) **(B)**, ascorbate peroxidase (APX) **(C)**, and superoxide dismutase (SOD) **(D)** enzymes after 13 days of treatment with 200 mM NaCl, 10 days of drought and 4 days of post-drought rewatering. The activity units are expressed as U⋅mg^−1^ protein. CAT activity was calculated by following the breakdown of H_2_O_2_. POD activity was calculated by following the guaiacol oxidation. APX activity was calculated by following the ascorbate oxidation. SOD activity was calculated by monitoring the superoxide radical-induced reduction of nitro blue tetrazolium (NBT). One unit of enzyme activity was defined as the amount of enzyme causing 50% inhibition per mg of protein. **(E)** Proline content, **(F)** Soluble sugar content. At least three biological replicates were included. Asterisks indicate significant differences between the transgenic and WT lines (*t*-test, *P < 0.05, **P < 0.01, ***P < 0.001).

The OE-2 and OE-7 lines exhibited a wilting phenotype earlier than the WT lines, with wilting appear at 10 d of drought (Figure 5A). At this time, a higher abundance of the blue precipitate derived from O_2_^–^ was obtained from the OE-2 and OE-7 lines than from the WT lines (Figure 5B), whereas a lower abundance of the brown precipitate derived from H_2_O_2_ was obtained from the OE-2 and OE-7 lines than from the WT lines (Figure 5C). The quantitative ROS assay showed results consistent with the *in vivo* localization, with more O_2_^–^ (Figure 5F) and less H_2_O_2_ (Figure 5E) produced in the OE-2 and OE-7 lines than in the WT plants. In addition, the OE-2, OE-7 and WT lines exhibited no significant phenotypic differences after 13 d of treatment with 200 mM NaCl, but more severe ROS production was found in the OE-2 and OE-7 lines than in the WT plants (Supplementary Figures S1A–E). Moreover, the MDA contents under both drought and NaCl stress conditions were significantly higher in the OE-2 and OE-7 lines than that in the WT plants (Figure 5G and Supplementary Figure S1F), which indicated a more severe oxidative damage in the transgenic lines. The determination of antioxidant enzymes activities showed that the OE-2 and OE-7 lines exhibited higher CAT activity than the WT plants under both drought and NaCl stress conditions (Figure 6A). The POD activities in the OE-2 and OE-7 lines were significantly higher than that of WT plants under drought and NaCl treatments (Figure 6B). No difference in APX activity was found among the OE-2, OE-7 and WT lines under drought and NaCl conditions (Figure 6C). However, the OE-2 and OE-7 lines exhibited significantly lower SOD activity than the WT plants under drought and NaCl conditions (Figure 6D). The proline and soluble sugar contents were substantially increased under drought and NaCl conditions in both the WT and transgenic lines, and significantly lower contents were obtained in the OE-2 and OE-7 lines than in the WT plants (Figures 6E,F), which indicated that *MaCDSP32* obstructs the accumulation of proline and soluble sugars in response to abiotic stress. Overall, the overexpression of *MaCDSP32* appears to increase sensitivity to drought and NaCl stress in transgenic tobacco.

We found that the OE-2 and OE-7 lines completely recovered after 4 d of rewatering, whereas the WT lines became severely wilted, and their leaves severely yellowed (Figure 5A). During drought stress, the proportion of wilted leaves was higher in the OE-2 and OE-7 lines than in the WT lines; however, it decreased markedly in the transgenic lines after rewatering, becoming lower than that in the WT plants (Figure 5D). Furthermore, the production of H_2_O_2_ and O_2_^–^ in the OE-2 and OE-7 lines was markedly decreased after rewatering compared with that under drought, whereas corresponding changes were not observed in the WT plants (Figures 5E,F). Decreases in MDA content were obtained with the production of ROS in the OE-2 and OE-7 lines, whereas MDA content increased continuously in WT plants (Figure 5G). In the WT plants, *MaCDSP32* expression was upregulated under drought and rewatering conditions compared with that under control conditions. In contrast, *MaCDSP32* expression in the OE-2 and OE-7 lines was strongly upregulated under drought conditions and slightly downregulated after rewatering (Figure 5H). The physiological parameters that significantly differed between the transgenic lines and WT plants in response to drought treatment were measured again after rewatering. Decreased CAT activity was obtained after 4 d of rewatering, but no significant difference was found among the OE-2, OE-7 and WT lines (Figure 6A). Decreased POD activity was also observed after rewatering, although significantly higher recovery of POD activity was observed in the OE-2 and OE-7 lines than in the WT plants (Figure 6B). After rewatering, significantly higher SOD activity was observed in the OE-2 and OE-7 lines than in the WT lines (Figure 6D). In addition, the proline and soluble sugar contents after rewatering were significantly higher in the transgenic lines than in the WT plants (Figures 6E,F). These differences suggested that *MaCDSP32* is involved in the bidirectional regulation of the associated physiological pathways in response to both drought stress and drought release in plants.

### *MaCDSP32* Affects Photosynthetic System Parameters Under Abiotic Stress

Because the expression of the *MaCDSP32* gene had a circadian rhythm and because the gene product was located in chloroplasts, parameters related to the photosynthetic system were detected under various abiotic stress treatments (10 d of drought, 13 d of NaCl and 25 h of MV) to assess the effects on photosynthetic efficiency. Both the two OE and WT lines exhibited severe wilting after 25 h of MV treatment, and no significant difference in phenotype was observed among the lines (Supplementary Figure S2F). In general, no significant difference in *Pn* values was found among the OE-2, OE-7 and WT lines under the drought, NaCl or oxidation conditions (Supplementary Figure S2A). Under the drought and oxidation conditions, stomatal conductance was lower in the OE-2 and OE-7 lines than in the WT plants (Supplementary Figure S2B). The intercellular CO_2_ concentration was significantly lower in the OE-2 and OE-7 lines than in WT under NaCl stress (Supplementary Figure S2C). Leaf temperature was significantly higher in the OE-2 and OE-7 lines than in the WT plants under the control and drought conditions (Supplementary Figure S2D). Interestingly, the results obtained for the transpiration rate were highly consistent with those obtained for the stomatal conductance under the stress treatments, and the OE-2 and OE-7 lines exhibited a significantly lower transpiration rates than the WT plants (Supplementary Figure S2E). These results indicated that *MaCDSP32* might affect the regulation of gas parameters under abiotic stress in plants. Additionally, after 13 d of NaCl treatment, the concentrations of *C*_*a*_, *C*_*b*_, total chlorophyll and carotenoids were significantly higher in the OE-2 and OE-7 lines than in the WT lines (Supplementary Figure S3). These results indicate that *MaCDSP32* might protect photosynthetic pigments under NaCl stress.

### *MaCDSP32* Enhances Seed Germination and Seedling Growth Under Osmotic Stress

**FIGURE 7:**
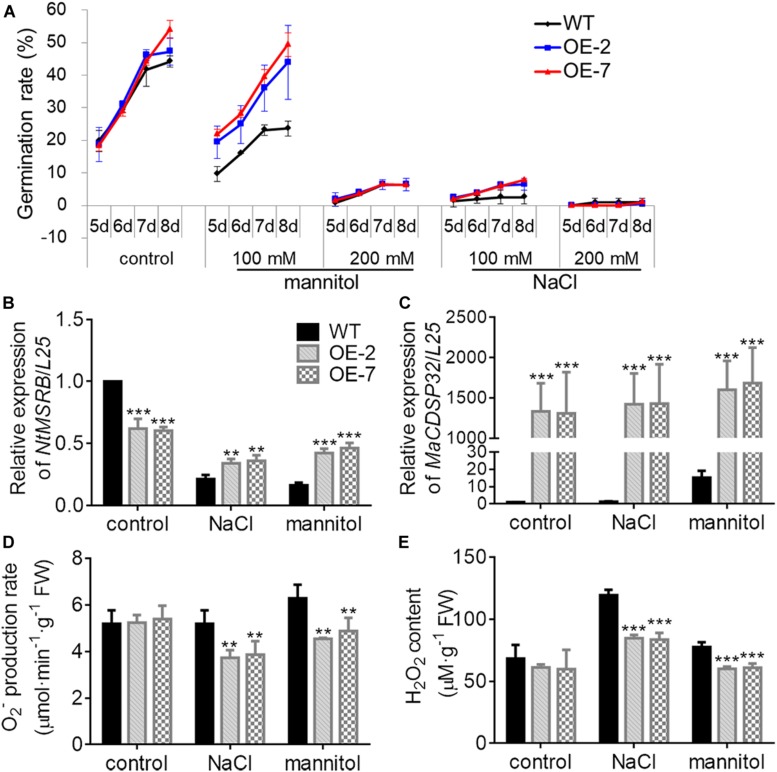
Overexpression of *MaCDSP32* enhances seed germination in transgenic tobacco under osmotic stress. **(A)** Seed germination rates after sowing at 5, 6, 7, and 8 days of treatment with mannitol or NaCl at various concentrations (100 mM and 200 mM). **(B,C)** Relative gene expression of *NtMSRB* **(B)** and *MaCDSP32* **(C)** in germinating seeds after 6 days of treatment with 100 mM NaCl or mannitol. **(D,E)** Production of O_2_^–^ **(D)** and H_2_O_2_ **(E)** after 6 days of treatment with 100 mM NaCl or mannitol. At least three biological replicates were included. Asterisks indicate significant differences between the transgenic and WT lines (*t*-test, **P < 0.01, ***P < 0.001).

**FIGURE 8:**
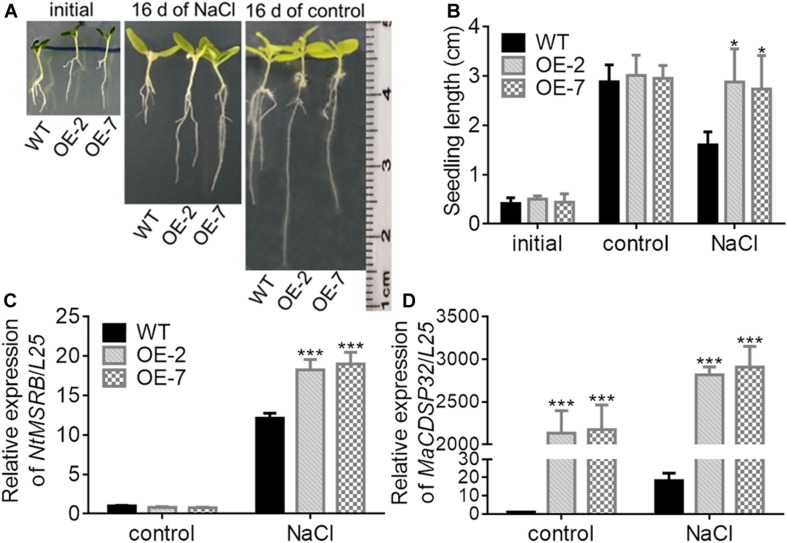
Overexpression of *MaCDSP32* enhances the growth of transgenic tobacco seedlings under NaCl stress. **(A)** Phenotypes of two transgenic lines of tobacco seedlings (OE-2 and OE-7) and of WT tobacco seedlings at transplant (initial), after 16 days of 200 mM NaCl stress and after 16 days under normal conditions. **(B)** Seedlings lengths of treated tobacco. **(C,D)** Relative expression of *NtMSRB* **(C)** and *MaCDSP32* **(D)** in tobacco seedlings after 16 days of 200 mM NaCl stress. Initial: newly transplanted seedlings, which germinated 9 days after sowing under normal conditions. At least three biological replicates were included. Asterisks indicate significant differences between the transgenic and WT lines (*t*-test, *P < 0.05, ***P < 0.001).

MSRB protein is considered a well-known target of CDSP32, and is involved in the regulation of seed viability, and the metabolites of methionine can facilitate seed germination (Catusse et al., 2011; Chatelain et al., 2013). Therefore, the seed germination of two transgenic tobacco OE lines were estimated in this study (Figure 7A). Under normal conditions, the germination rates of WT, OE-2 and OE-7 seeds at the early stage were not significantly different. However, by day 8, the germination rates of the OE-2 (47.29%) and OE-7 seeds (54.03%) were markedly higher than that of the WT seeds (44.15%). Under exposure to 100 mM mannitol or 100 mM NaCl, the higher germination capacity of OE-2 and OE-7 seeds than of WT seeds was more pronounced. Germination was suppressed in the OE-2, OE-7, and WT seeds during exposure to a high NaCl or mannitol concentration (200 mM). The expression of *NtMSRB* in germinating seeds was downregulated under 100 mM NaCl or mannitol treatment compared with the control expression, but was higher in the OE-2 and OE-7 seeds than in the WT seeds (Figure 7B). The expression of *MaCDSP32* in germinating seeds was upregulated under 100 mM NaCl or mannitol treatment compared with the control expression level, and was significantly higher in the seeds of the OE-2 and OE-7 lines those that of the WT lines (Figure 7C). H_2_O_2_ and O_2_^–^ levels were significantly lower in the germinating seeds of the OE-2 and OE-7 lines than in those of the WT lines under 100 mM mannitol or NaCl treatment (Figures 7D,E). These results revealed that *MaCDSP32* enhanced the seed germination rate under osmotic stress. Regarding seedling growth no significant difference was found between the two OE and WT seedlings at the initial time point or after 16 d of growth under control condition; however, after 16 days of growth under 200 mM NaCl treatment, the lengths of OE-2 and OE-7 seedlings were significantly greater than the length of WT seedlings (Figures 8A,B). Furthermore, after 16 d of NaCl treatment, the expression of *NtMSRB* was upregulated relative to control levels in the seedlings of both the OE and WT genotypes and was significantly higher in the OE-2 and OE-7 seedlings than in the WT seedlings (Figure 8C). In addition, *MaCDSP32* expression was upregulated under NaCl treatment and was significantly higher in the OE-2 and OE-7 seedlings than in the WT seedlings (Figure 8D). These results revealed that *MaCDSP32* potentially strengthens seedling growth under NaCl stress and might participate in regulating the expression of *NtMSRB*.

## Discussion

A previous study revealed that CDSP32 protein was synthesized extensively under water deficit conditions (Rey et al., 1998), particularly under severe conditions (Broin et al., 2000). Here, we demonstrate that *MaCDSP32* is a drought-induced gene in mulberry that is mainly expressed in mature leaves rather than young leaves, old leaves, stems or petioles. Furthermore, MaCDSP32 is localized to the chloroplasts, and we deduced that *MaCDSP32* was involved in photosynthesis. In addition, the expression of *MaCDSP32* within a photoperiod was regular, and higher expression was detected during the middle of the day; these findings indicated that *MaCDSP32* is a circadian-regulated gene and closely related to photosynthetic light reactions.

Under dehydration, *MaMAPK* and *MaDREB1* were more strongly downregulated in *Inst* than in WT*_*Ma*_*, which indicated a negative correlation between *MaMAPK*/*MaDREB1* and *MaCDSP32* under water deficit conditions. The MAPK cascade and DREB1 pathway are involved in the transmission of stress signals and can activate the expression of stress tolerance genes (Sakuma et al., 2002; Choudhury et al., 2017). In general, plants respond to a variety of environmental constraints via orchestrated signaling events that coordinate modifications to transcriptional profiles, physiological processes and redox homeostasis. *MaCDSP32* might reduce drought tolerance through stress signal transduction by inhibiting the expression of *MaMAPK* and *MaDREB1*. Additionally, the transgenic *Inst* (*Inst*-1 and *Inst*-2) leaves and OE (OE-2 and OE-7) lines showed higher water loss rate or an earlier wilting phenotype than did the WT plants under drought and NaCl stress. Lower H_2_O_2_ production and higher O_2_^–^ production were found in transgenic tobacco lines than in the WT plants under both stress conditions, which indicated that *MaCDSP32* is involved in ROS metabolism in response to abiotic stress. *MaCDSP32* is likely to promote the removal of H_2_O_2_ but not O_2_^–^ under stress for the following reasons: Trxs participate in the sulfide oxidation by reducing H_2_O_2_ in cells (Broin et al., 2002), and MaCDSP32 is a Trx-like protein that has an active center similar to the functional domain of other Trxs. Moreover, larger amounts of MDA were detected in the transgenic tobacco lines than in the WT lines under stress conditions, which indicated more severe membrane lipid peroxidation damage in the former due to *MaCDSP32* overexpression. Significantly lower SOD activity was found in the transgenic lines than in the WT lines under the stress conditions, suggesting inefficient O_2_^–^ metabolism in the transgenic lines and consistent with the higher O_2_^–^ production observed in these lines. In addition, the concentrations of proline and soluble sugars were significantly lower in the transgenic lines than in the WT plants under stress conditions. We propose that *MaCDSP32* is likely a negative regulatory factor for the accumulation of these two osmotic substances under abiotic stress. The overexpression of *MaCDSP32* disrupts the redox equilibrium and the regulation of cellular turgor in response to abiotic stress. Notably, the opposite results were obtained after rewatering. Transgenic tobacco lines showed complete post-drought recovery, whereas the WT plants did not. The OE-2 and OE-7 lines exhibited higher SOD activity, increased proline and soluble sugar contents, and substantially lower detectable H_2_O_2_ and O_2_^–^ contents after rewatering compared with the WT plants. Downregulated *MaCDSP32* expression was obtained after rewatering compared with during drought in both OE lines, but the expression of *MaCDSP32* remained upregulated in the WT plants. This result suggests that the drought response mechanism involves *MaCDSP32*, which might antagonize the mechanism of osmotic pressure regulation in the cell, and that in the ROS scavenging pathway, *MaCDSP32* may be functionally redundant with the enzyme system and antioxidant enzyme system.

We deduced that *MaCDSP32* participates in multiple regulatory mechanisms by inhibiting or promoting antioxidant activity and the accumulation of osmotic-adjustment substances under abiotic stress so that plants can endure changes in the environment. We conjecture that the intricate regulatory network enmeshing *MaCDSP32* is responsible for the rapid wilting of the transgenic lines during severe water deficit and their maintenance of the minimum water consumption, which allows their survival under drought conditions. The SOD enzyme is the main site of the conversion of superfluous O_2_^–^ via its disproportionation in the chloroplast (Chen et al., 2018). Based on the results obtained for SOD activity and O_2_^–^ production demonstrate that *MaCDSP32* plays a key role in the regulation of ROS homeostasis by particularly targeting the SOD enzyme. Under drought conditions, *MaCDSP32* causes a decrease in SOD activity. A previous study showed that most Trxs are involved in the regulation of antioxidant enzyme activity (Arnér and Holmgren, 2000), and each member interacts with a specific antioxidant (Mata-Perez and Spoel, 2018). For instance, Trx-*y* specifically affects the activity of glutathione peroxidase (Navrot et al., 2006). These findings provide strong evidence supporting our conjecture that SOD is a target of *MaCDSP32*. Additionally, previous studies have revealed that CDSP32 is involved in osmoregulation (Pruvot et al., 1996) and that the accumulation of osmotic substances can enhance tolerance to stress by increasing cell turgor in plants (Ashraf and Foolad, 2007; Boriboonkaset et al., 2013). However, the compound that serves as the substrate is unclear. Our results suggest that proline and soluble sugars are two types of potential substrates of *MaCDSP32*.

ROS act as signaling molecules involved in the biological processes of plants under favorable conditions, whereas excess ROS can cause oxidative damage under adverse conditions (Mittler, 2017; Schneider et al., 2018). With a similar bimodality to that of ROS, *MaCDSP32* overexpression attenuates the tolerance of plants during drought stress and promotes post-drought recovery. Other events also arise from the same cause but have completely opposite results. A previous study revealed that P53 protein plays completely different roles in animal cells at 40°C and 43°C by promoting cell survival and apoptosis, respectively, because these different temperatures lead to different P53 protein stabilization mechanisms and activate different signaling pathways (Gong et al., 2016). Similarly, transgenic lines overexpressing *MaCDSP32* might have altered tolerance thresholds to mild and severe drought stress and show differences post-drought rewatering. Plants exhibit two types of drought tolerance: tolerance during drought and resilience after drought (Tyree et al., 2002). We hypothesize that the drought tolerance of transgenic tobacco involving *MaCDSP32* mainly belongs to the latter type, which is similar to the drought tolerance pattern of resurrection plants.

The expression of CDSP32 is regulated by posttranscriptional events, which are closely related to the plant developmental stage and environmental conditions (Broin et al., 2003). In addition, CDSP32 expression is mainly induced under severe stress conditions; a high abundance of CDSP32 in transgenic overexpression plants under mild stress conditions could disturb the plastidic redox state and might divert the reducing power (or electron transport direction) necessary for other protection systems (Broin et al., 2002). Therefore, we hypothesize that although the activities of antioxidative enzymes in *MaCDSP32*-overexpressing transgenic tobacco plants were quite similar under the control and rewatering conditions, the feedback responses in the plants differed among the different physiological and environmental conditions. The reducing power provided by the products of *MaCDSP32* expression was diverted and reallocated, which led to a different distribution of the *MaCDSP32*-related upstream and downstream processes involved in ROS homeostasis regulation and thereby resulted in differences in superoxide production. Furthermore, the specific regulatory mechanism induced by severe drought stress in transgenic tobacco plants is one of the possible reasons for the reduced production of O_2_^–^. This process might involve a complex mechanism, and further exploration and verification are needed.

Under NaCl stress, transgenic tobacco lines maintain higher chlorophyll and carotenoid contents than the WT, which suggested that *MaCDSP32* plays a positive role in protecting chloroplast photosynthetic pigments against NaCl stress. This result indicated that *MaCDSP32* might affect the activity of chlorophyll-related enzymes under NaCl stress. Although NaCl treatment can increase the activity of chlorophyllase, the synthesis and degradation of chlorophyll are mainly affected by the activity of chlorophyll enzymes (Chen et al., 2018). This increased chlorophyllase activity might induce CDSP32 activity, which functions to protect photosynthetic system elements (Broin et al., 2002). Moreover, *MaCDSP32* enhanced seed germination and seedling growth in transgenic tobacco under NaCl and mannitol stress. Because MSRB participates involved in the regulation of seed vitality by reducing oxidized methionine (Laugier et al., 2010; Chatelain et al., 2013), we suggest that *MaCDSP32* and its target *NtMSRB* work together to reduce the production of H_2_O_2_ and O_2_^–^ and facilitate seed germination. We propose that the *MaCDSP32*/*NtMSRB* reduction system plays an important role in the effective scavenging of ROS produced under osmotic stress.

## Conclusion

**FIGURE 9:**
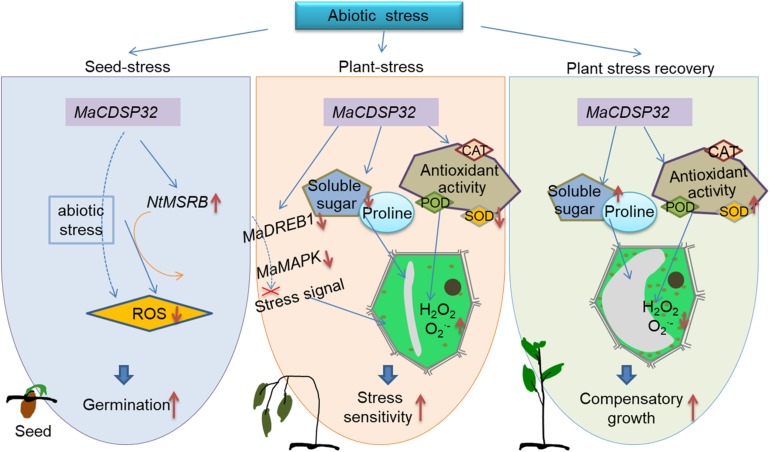
Schematic of the contribution of *MaCDSP32* to plant drought tolerance through the regulation of redox metabolism, signal transduction and osmotic pressure. The solid orange arrows represent findings from the literature; the solid blue arrows represent finding of the present study; the dashed blue arrows represent our speculations based on the findings of the present study; the red bold solid down arrows indicates downregulation; the red bold solid up arrows indicates upregulation; and the red cross mark indicates inhibition.

The present study provides novel insights into the functions of *MaCDSP32* in plants. A schematic that incorporates the abiotic stress response mechanism mediated by *MaCDSP32* is shown in Figure 9. *MaCDSP32* overexpression promotes the gene expression of *NtMSRB*, which enhances seed germination under stress conditions. *MaCDSP32* influences ROS production by regulating the expression of *MaDREB1* and *MaMAPK*, the accumulation of soluble sugars and proline and the activities of antioxidant enzymes (especially POD and SOD) to affect the resistance of transgenic plants.

Due to the rapid development of human civilization, plants inevitably face increasingly serious environmental constraints. Plants with stronger vitality and adaptability are urgently needed for future plant and crop breeding. The abundant resources available in nature, such as the resurrection plant *Selaginella tamariscina*, might lead to enlightenment. Under water deficit conditions, these desiccation-tolerant species maintain significantly higher levels of soluble sugars, amino acids and other metabolites, which help the detoxification of excess ROS and contribute to desiccation tolerance (Xu et al., 2018). Our study suggests that *MaCDSP32* positively contributes to the post-drought recovery strategy and repair mechanisms of plants, and these effects give plants the ability to survive water stress and enhance the plant survival rate after stress relief.

## Data Availability Statement

The raw data supporting the conclusions of this article will be made available by the authors, without undue reservation, to any qualified researcher.

## Author Contributions

HS and FJ conceived the original research plans, performed most of the experiments, analyzed the data, and wrote the manuscript. WZ, HL, CS, and YQ provided much assistance in the sampling and data analysis stage. All the authors reviewed and interpreted the data, edited the manuscript, reviewed the results, and approved the final version of the manuscript.

## Conflict of Interest

The authors declare that the research was conducted in the absence of any commercial or financial relationships that could be construed as a potential conflict of interest.

## Supplementary Material

FIGURE S1 Production of reactive oxygen species (ROS) in tobacco under NaCl stress. **(A)** Phenotypes of 3-month-old WT and transgenic (OE-2 and OE-7) tobacco lines under 13 days of treatment with 200 mM NaCl. **(B,C)** *In vivo* histochemical detection of O_2_^–^ **(B)** and H_2_O_2_ **(C)** in plants under treatment. **(D,E)** Quantification of H_2_O_2_ **(D)**, O_2_^–^ **(E)**, and **(F)** MDA contents. At least three biological replicates were included. Asterisks indicate significant differences between the transgenic and WT lines (*t*-test, ^∗^*P* < 0.05, ^∗∗∗^*P* < 0.001).

FIGURE S2 Gas exchange parameters in transgenic tobacco under abiotic stress. **(A)** Net photosynthetic rate (*Pn*). **(B)** Stomatal conductance. **(C)** Intercellular CO_2_ concentration. **(D)** Leaf temperature. **(E)** Transpiration rate. **(F)** Phenotypes of 3-month-old WT and transgenic (OE-2 and OE-7) tobacco lines treated with 10 μM methyl viologen (MV) for 25 h. The following treatments were used: 10 days of soil drought, 13 days of treatment with 200 mM NaCl and 25 h of treatment with 10 μM MV. At least three biological replicates were included. Asterisks indicate significant differences between the transgenic and WT lines (*t*-test, ^∗^*P* < 0.05, ^∗∗^*P* < 0.01 and ^∗∗∗^*P* < 0.001).

FIGURE S3 Overexpression of *MaCDSP32* helps maintain chloroplast pigment levels in transgenic tobacco under NaCl stress. **(A)** Total chlorophyll content. **(B)** Chlorophyll *a* content. **(C)** Chlorophyll *b* content. **(D)** Carotenoids content. Three-month-old WT and transgenic (OE-2 and OE-7) tobacco lines were treated with 200 mM NaCl for 13 days. At least three biological replicates were included. Asterisks indicate significant differences between the transgenic and WT lines (*t*-test, ^∗∗^*P* < 0.01).
