# Supplementary figures and images for: MaCDSP32 From Mulberry Enhances Resilience Post-drought by Regulating Antioxidant Activity and the Osmotic Content in Transgenic Tobacco
Source: Front Plant Sci. 2020 Apr 16;11:419. doi: 10.3389/fpls.2020.00419 (PMC7177052; doi:10.3389/fpls.2020.00419)

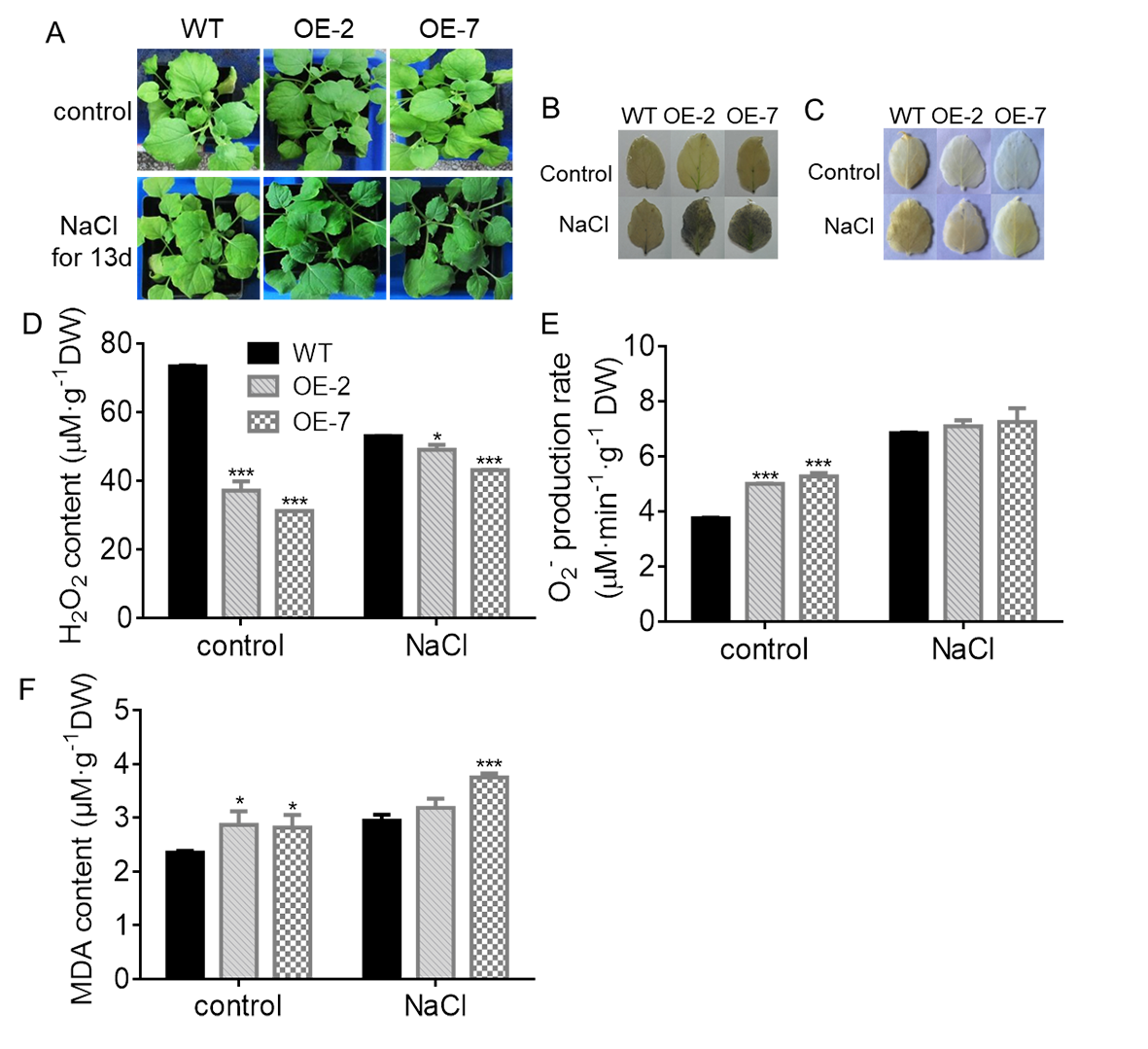

Supplement: FIGURE S1 — Production of reactive oxygen species (ROS) in tobacco under NaCl stress. (A) Phenotypes of 3-month-old WT and transgenic (OE-2 and OE-7) tobacco lines under 13 days of treatment with 200 mM NaCl. (B,C) In vivo histochemical detection of O2– (B) and H2O2 (C) in plants under treatment. (D,E) Quantification of H2O2 (D), O2– (E), and (F) MDA contents. At least three biological replicates were included. Asterisks indicate significant differences between the transgenic and WT lines (t-test, ∗P < 0.05, ∗∗∗P < 0.001). [file Image_1.TIF]

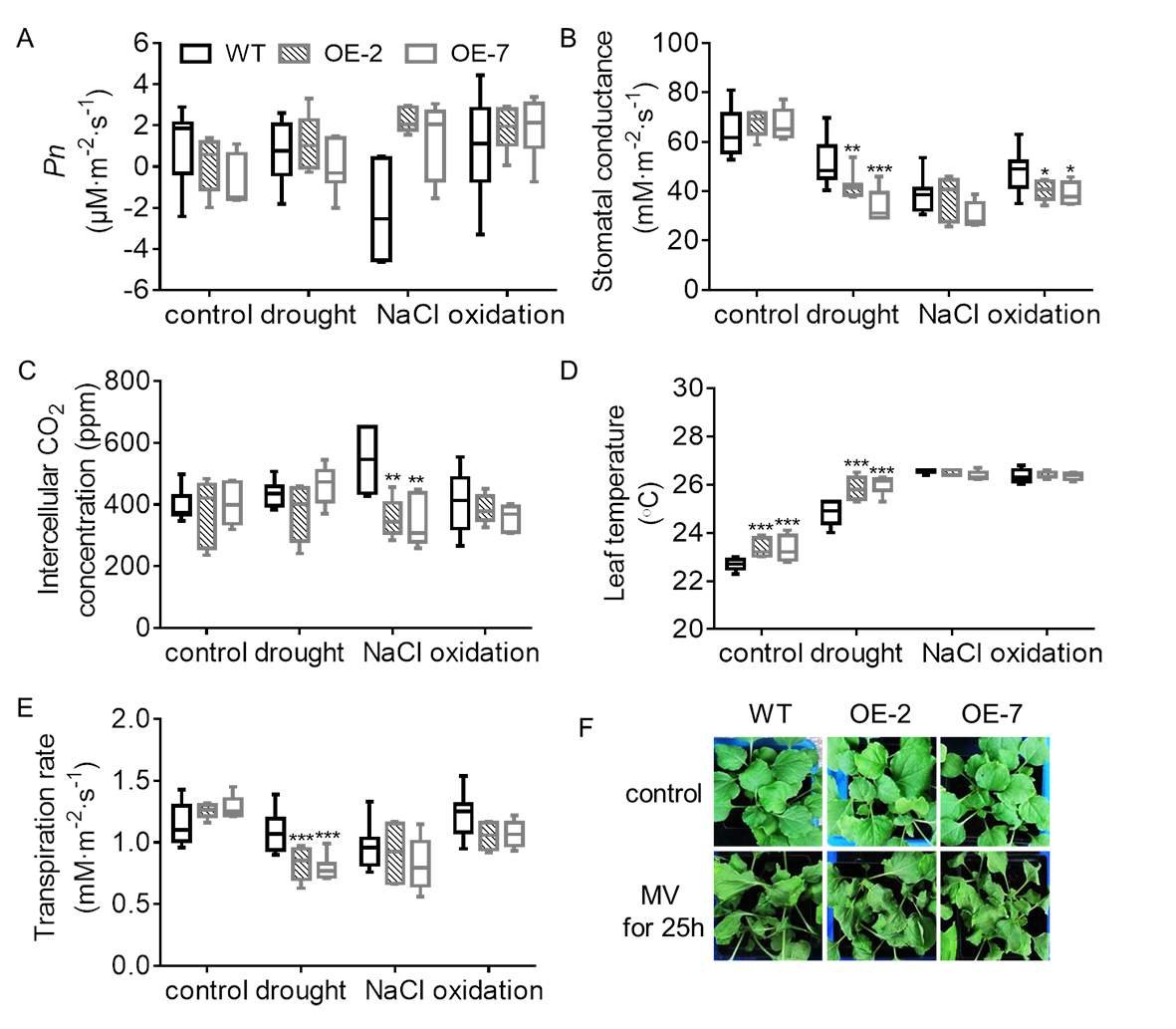

Supplement: FIGURE S2 — Gas exchange parameters in transgenic tobacco under abiotic stress. (A) Net photosynthetic rate (Pn). (B) Stomatal conductance. (C) Intercellular CO2 concentration. (D) Leaf temperature. (E) Transpiration rate. (F) Phenotypes of 3-month-old WT and transgenic (OE-2 and OE-7) tobacco lines treated with 10 μM methyl viologen (MV) for 25 h. The following treatments were used: 10 days of soil drought, 13 days of treatment with 200 mM NaCl and 25 h of treatment with 10 μM MV. At least three biological replicates were included. Asterisks indicate significant differences between the transgenic and WT lines (t-test, ∗P < 0.05, ∗∗P < 0.01 and ∗∗∗P < 0.001). [file Image_2.TIF]

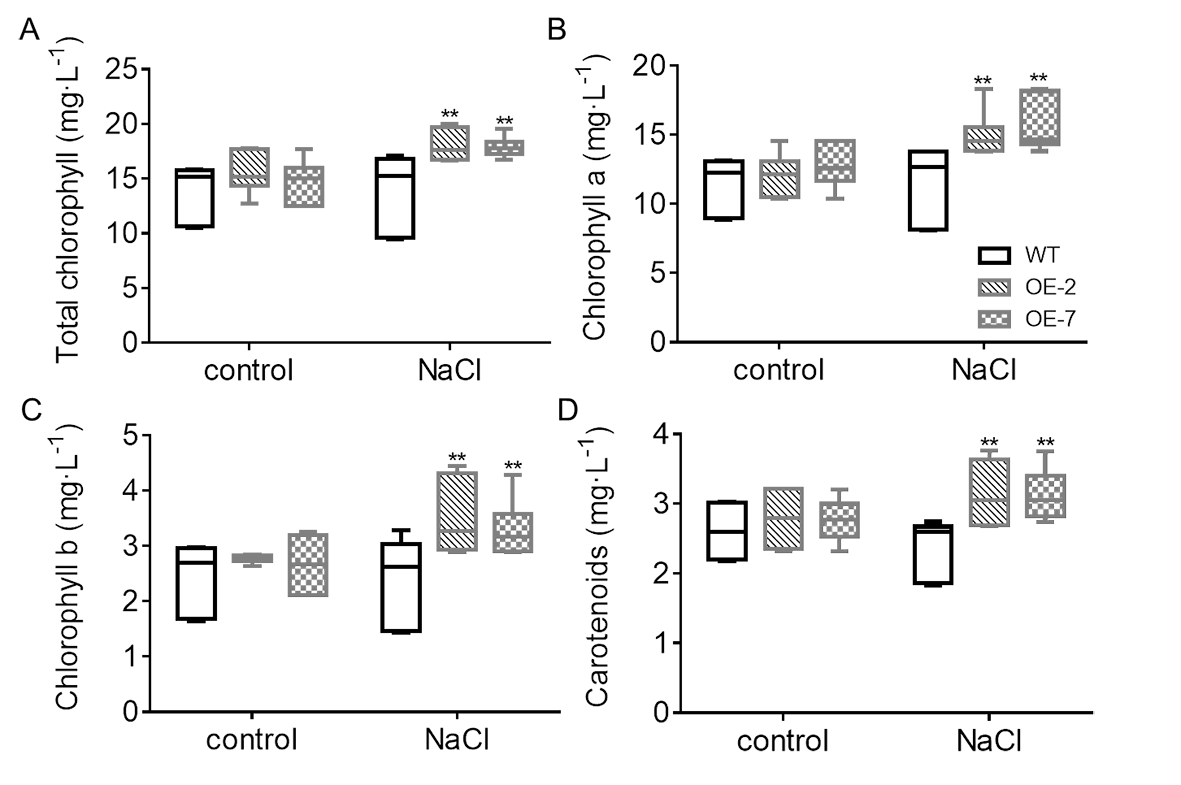

Supplement: FIGURE S3 — Overexpression of MaCDSP32 helps maintain chloroplast pigment levels in transgenic tobacco under NaCl stress. (A) Total chlorophyll content. (B) Chlorophyll a content. (C) Chlorophyll b content. (D) Carotenoids content. Three-month-old WT and transgenic (OE-2 and OE-7) tobacco lines were treated with 200 mM NaCl for 13 days. At least three biological replicates were included. Asterisks indicate significant differences between the transgenic and WT lines (t-test, ∗∗P < 0.01). [file Image_3.TIF]
