# Supplementary material for: MaCDSP32 From Mulberry Enhances Resilience Post-drought by Regulating Antioxidant Activity and the Osmotic Content in Transgenic Tobacco
Source: Front Plant Sci. 2020 Apr 16;11:419. doi: 10.3389/fpls.2020.00419 (PMC7177052; doi:10.3389/fpls.2020.00419)
Supplement: Supplementary file 4 [file Table_1.DOCX]

**Supplement Table S1** Primers used in this study

| Genes | Primers (5’-3’) | Annotation |
| --- | --- | --- |
| *MaCDSP32*  (full-length) | Forward: ATGGCTACAATCACAAACTTTC  Reverse: ATACGTCACACGAACCCCTTGG | For cloning |
| *MaCDSP32*  (Plasmid) | Forward: CACGGGGGACGAGCTCGGGTACCATGGCTACAATCACA  AACTTTCTACC  Reverse: CCATGGTGTCGACTCTAGAATACGTCACACGAACCCCTTG  GTAT | For homologous recombination |
| *MaCDSP32* | Forward: CGTCGGGTTGAAGCATTG  Reverse: GTCGCCGTTCATCCTTGC | For qRT-PCR |
| *MaACTIN* | Forward: GAGCAAGGAGATCACAGCCC  Reverse: CCAGACTCGTCGTACTCGC |  |
| *MaMAPK* | Forward: AGCACTCCAACACCCTT  Reverse: CCACATCATCTCCCTTATC |  |
| *MaDREB1* | Forward: TGTATGAAGGGAAAGGGAGG  Reverse: TCAATGGCTGTTGGGAAGGT |  |
| *NtMSRB* | Forward: AAAGAATGCGGTACAAACTTCAG  Reverse: ATCAACGGCGGAGAAAGATAG |  |
| *NtL25* | Forward: CCCCTCACCACAGAGTCTGC  Reverse: AAGGGTGTTGTTGTCCTCAATCTT |  |
